# Unusual Ebola Virus Chain of Transmission, Conakry, Guinea, 2014–2015

**DOI:** 10.3201/eid2212.160847

**Published:** 2016-12

**Authors:** Mory Keita, Sophie Duraffour, Nicholas J. Loman, Andrew Rambaut, Boubacar Diallo, Nfaly Magassouba, Miles W. Carroll, Joshua Quick, Amadou A. Sall, Judith R. Glynn, Pierre Formenty, Lorenzo Subissi, Ousmane Faye

**Affiliations:** World Health Organization, Conakry, Guinea (M. Keita, B. Diallo);; Bernhard Nocht Institute for Tropical Medicine, Hamburg, Germany (S. Duraffour);; The European Mobile Laboratory Consortium, Hamburg (S. Duraffour, M.W. Carroll);; University of Birmingham, Birmingham, UK (N.J. Loman, J. Quick);; University of Edinburgh, Edinburgh, Scotland, UK (A. Rambaut);; Université Gamal Abdel Nasser de Conakry, Conakry (N. Magassouba);; Public Health England, Porton Down, Salisbury, UK (M.W. Carroll);; University of Southampton South General Hospital, Southampton, UK (M.W. Carroll);; Institut Pasteur, Dakar, Senegal (A.A. Sall, O. Faye);; London School of Hygiene and Tropical Medicine, London, UK (J.R. Glynn);; World Health Organization, Geneva, Switzerland (P. Formenty);; European Centre for Disease Prevention and Control/World Health Organization, Conakry (L. Subissi)

**Keywords:** Ebola, Ebola virus disease, Ebola virus, molecular evolution, transmission, flare-up, viruses, Guinea

## Abstract

In October 2015, a new case of Ebola virus disease in Guinea was detected. Case investigation, serology, and whole-genome sequencing indicated possible transmission of the virus from an Ebola virus disease survivor to another person and then to the case-patient reported here. This transmission chain over 11 months suggests slow Ebola virus evolution.

As of May 6, 2016, a total of 28,616 Ebola virus disease (EVD) cases, including 11,310 deaths, had been reported since December 2013, mainly from Guinea, Sierra Leone, and Liberia (*1*). Ebola virus often persists in immune-privileged sites of survivors, resulting in detectable virus in semen and other body fluids (*2*–*5*). Because sperm can shed Ebola virus (EBOV) for <10 months after illness onset, exposure to semen of infected survivors can lead to EVD flare-ups (*5*–*8*). In utero transmission of EBOV from an asymptomatic mother is also possible (*9*).

A powerful tool for following the course of an epidemic of diseases for which human-to-human transmission is prevalent is molecular typing. In Guinea, an in-field sequencing facility was deployed in May 2015, and by September, when the outbreak was fading, EBOV genomes were available for ≈90% of new EVD cases (*10*). We report epidemiologic and molecular evidence of an unusual chain of EBOV transmission. This chain includes an EVD survivor, who recovered in December 2014; possibly his wife (the woman) or a third unknown person; and the woman’s brother, who became ill with EVD in October 2015 (the case-patient). 

The National Committee of Ethics in Medical Research of Guinea approved use of archived diagnostic samples and corresponding patient data for this study (permit no. 11/CNERS/14). Written consent for publication of confidential data was collected for all patients described here.

## The Study

On October 8, 2015, the case-patient in Conakry, Guinea, became ill with fever and appetite loss. His brother-in-law, an EVD survivor and nurse living in the same household, diagnosed malaria, but the case-patient did not respond to malaria treatment; diarrhea, headache, and abdominal pain developed. Four days later, he started bleeding from the nose. On October 13, reverse transmission PCR (RT-PCR) of the case-patient’s blood was positive for EBOV (*11*). 

From September 2015 until confirmation of EVD for this case-patient, only 7 cases of EVD in Guinea had been reported, all part of the same chain of transmission. Of these cases, virus was not sequenced for 2 because the epidemiologic link was not clearly established. Because the case-patient we report did not have any link with ongoing EVD clusters investigated in Guinea or Sierra Leone, the most likely source of infection was the survivor. Because EBOV is shed in survivor’s semen long after recovery (*7*), within-marriage sexual transmission from the survivor to the woman was inferred. 

**Figure 1:**
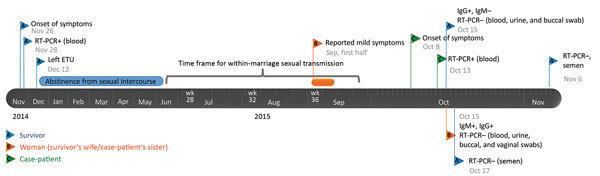
Timeline of the reported chain of transmission of Ebola virus involving 3 persons in Conkary, Guinea, 2014–2015. ETU, Ebola treatment unit; RT-PCR, reverse transcription PCR; –, negative; +, positive.

Blood, urine, and buccal swab samples from the couple were collected, together with semen and vaginal swab samples; all were negative for EBOV by RT-PCR. Blood, urine, buccal, and vaginal swab samples were processed as in (*11*) and seminal fluid samples as in (*10*). The woman’s serum was positive according to IgG and IgM ELISA testing (both titers >1:6,400); the survivor’s serum was positive for IgG only (titer >1:3,200) (*12*). Serum from the only other household member, the couple’s 11-year-old daughter, was negative for IgG and IgM. Although the woman’s IgM titer was suggestive of a recent EBOV infection, she reported having had only a mild 2-day clinical episode back in early September, characterized by myalgia, joint pain, and low-back pain (Figure 1). The couple declared that they had had no sexual intercourse for 6 months after the survivor was discharged from an Ebola treatment unit. 

**Figure 2:**
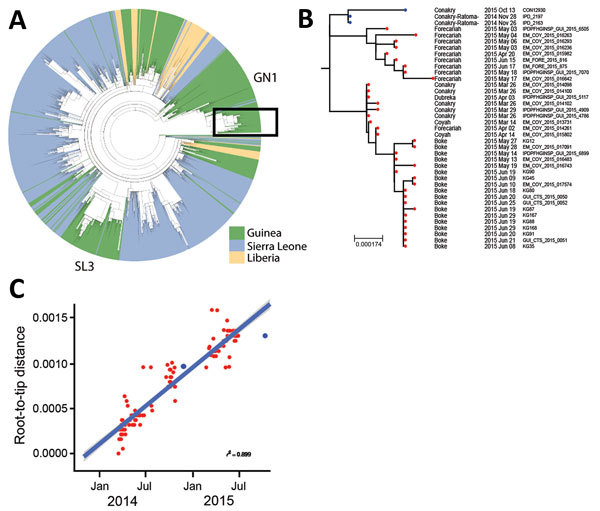
Phylogenetic analysis of Ebola viruses. A) Maximum-likelihood tree of 1,067 Ebola virus sequences from the West Africa epidemic, showing country of origin of each isolate. The 2 lineages that circulated in Guinea in 2015 were GN1 and SL3. Ebola virus disease cases from Conakry, Guinea, obtained in the second half of 2015 (from June on) are denoted by a red dot on the tree branches. Box indicates 2015 isolates from the GN1 lineage. B) Expanded view of the GN1 lineage from panel A. Blue dots indicate sequences from cases described in this article (survivor, IPD_2197; person who infected the survivor, IPD_2163; case-patient, CON12930); red dots indicate other cases from 2015. Sequences can be found under the European Nucleotide Archive study no. ERP011834 (http://www.ebi.ac.uk/ena/data/view/PRJEB10571). Scale bar indicates nucleotide substitutions per siite. C) Root-to-tip plot for the entire GN1 lineage; colors match those in panel B. A linear model line of best fit is shown; gray shading represents the confidence level around the regression line as SE.

The near full-genome sequence of virus from the case-patient, obtained as previously described (*10*), was available on October 15, 2015. Phylogenetic analysis demonstrated that it belonged to the GN1 lineage rather than the SL3 lineage that was circulating in Conakry during the second half of 2015 (Figure 2, panel A). Thus, the cases were probably not linked to the known ongoing chain of transmission in this area because sequencing of the viruses involved in the Guinea epidemic had been thoroughly performed at this time and no GN1 lineage viruses had been detected in Guinea since the end of June 2015 (*10*). The sequence of the virus isolated from the case-patient, however, could not be closely linked to any known subclusters within GN1. The most closely related viruses were recovered from EVD patients in March 2015, but phylogenetic inference suggests that the new sequence had not evolved from these patients and instead shared a common ancestor from late 2014 (Figure 2, panel B).

Because various samples from the survivor (including semen) and from the woman were negative for EBOV (Figure 1), serum collected during the survivor’s acute phase of EVD (November 28, 2014) was retrieved from an archived collection and sequenced, together with a sample from the person who was thought to have transmitted the infection to him (IPD_2163) (Figure 2, panel B). These 2 EBOV sequences were indistinguishable and clustered with virus sequences from the case-patient, differing at 6 nt sites. This difference over an 11-month period is smaller than expected from the estimated EBOV evolutionary rate that would predict 22.5 (95% CI 13–33) mutations (Figure 2, panel C).

Several lines of evidence converge toward an unusual chain of transmission. Most likely, the first patient sexually transmitted EBOV to the woman 9 months after his onset of EVD in 2014, and then either the woman (with a recent history of undetected EBOV infection) or a third unknown person transmitted the virus to the case-patient. After 10.5 months, virus from the case-patient differed from that of the survivor by only 6 nt substitutions. These substitutions were not present in other virus sequences from the GN1 lineage, demonstrating that the isolates are closely related to each other and more distantly related to others.

The number of substitutions between the 2 genomes was 3.7 times lower than would be expected after human-to-human transmission and may indicate virus persistence in 1 person. This hypothesis is in line with reduced evolutionary rates of persistent EBOV reported in Liberia and Sierra Leone (*7*,*8*,*13*).

Serologic data revealed that the woman had recently been infected with EBOV. However, none of her samples (i.e., blood, urine, and vaginal swab) were positive for EBOV by RT-PCR; therefore, no genomic data were available.

The serial interval between the case-patient’s onset of symptoms and that of the woman (≈30 days) is longer than the usual EVD maximum incubation period (21 days). Therefore, the woman may have more recently experienced mild or asymptomatic infection.

We have no molecular evidence that the woman was part of this chain of transmission; it is plausible that a third unknown person infected the case-patient. It was impossible to investigate potential direct male-to-male sexual transmission.

## Conclusions

A likely explanation of our observations, integrating genomic, serologic, and epidemiologic data, is that within-marriage sexual transmission occurred (inferred from the positive IgM titers of the woman) and then the woman infected her brother through close contact. They all lived in the same small household in poor hygienic conditions; shared toilets, meals, and at times beds; and cared for each other. Alternatively, a third unknown person may have been the link in the transmission from the survivor to the case-patient.

Interim advice with regard to sexual transmission of EBOV has been updated recently (*14*). All EVD survivors and their sex partners should receive condoms and counseling to ensure safer sex practices. Safer sex practices should continue until the results of semen testing are negative twice or, if testing is unavailable, for 12 months. The safer sex strategy and testing could be complemented by new medical countermeasures that need to be assessed and include use of antiviral drugs, vaccination of relatives and sex partners of survivors, or both.
